# Understanding the Composition, Biosynthesis, Accumulation and Transport of Flavonoids in Crops for the Promotion of Crops as Healthy Sources of Flavonoids for Human Consumption

**DOI:** 10.3390/nu12061717

**Published:** 2020-06-08

**Authors:** Yee-Shan Ku, Ming-Sin Ng, Sau-Shan Cheng, Annie Wing-Yi Lo, Zhixia Xiao, Tai-Sun Shin, Gyuhwa Chung, Hon-Ming Lam

**Affiliations:** 1 Centre for Soybean Research of the State Key Laboratory of Agrobiotechnology and School of Life Sciences, The Chinese University of Hong Kong, Hong Kong, China; ysku@ymail.com (Y.-S.K.); sammingsin0212@gmail.com (M.-S.N.); chengsaushan@yahoo.com (S.-S.C.); wingyilo@cuhk.edu.hk (A.W.-Y.L.); obennoname@gmail.com (Z.X.); 2 Division of Food and Nutrition, Chonnam National University, Gwangju 61186, Korea; shints@chonnam.ac.kr; 3 Department of Biotechnology, Chonnam National University, Yeosu 59626, Korea; 4 Shenzhen Research Institute, The Chinese University of Hong Kong, Shenzhen 518000, China

**Keywords:** crops, phenolic compounds, flavonoids, biosynthesis pathway, ABC transporters, MATE transporters, nutrition, health

## Abstract

Flavonoids are a class of polyphenolic compounds that naturally occur in plants. Sub-groups of flavonoids include flavone, flavonol, flavanone, flavanonol, anthocyanidin, flavanol and isoflavone. The various modifications on flavonoid molecules further increase the diversity of flavonoids. Certain crops are famous for being enriched in specific flavonoids. For example, anthocyanins, which give rise to a purplish color, are the characteristic compounds in berries; flavanols are enriched in teas; and isoflavones are uniquely found in several legumes. It is widely accepted that the antioxidative properties of flavonoids are beneficial for human health. In this review, we summarize the classification of the different sub-groups of flavonoids based on their molecular structures. The health benefits of flavonoids are addressed from the perspective of their molecular structures. The flavonoid biosynthesis pathways are compared among different crops to highlight the mechanisms that lead to the differential accumulation of different sub-groups of flavonoids. In addition, the mechanisms and genes involved in the transport and accumulation of flavonoids in crops are discussed. We hope the understanding of flavonoid accumulation in crops will guide the proper balance in their consumption to improve human health.

## 1. Introduction

With the improved awareness of nutrition and health worldwide, the consumption of health supplements has been ever increasing. Crops, including a variety of fruits, vegetables and legumes, are rich in flavonoids, which are known to exhibit antioxidative and antimicrobial activities. In this review, we address the potential health benefits, and the storage and transport, of flavonoids at the molecular level. Understanding how flavonoids can provide health benefits will enable the use of crops as health supplements, while the knowledge of the molecular mechanisms regulating the intra- and extra-cellular storage and transport of flavonoids in plants will facilitate breeding programs to improve their contents in crops.

## 2. Classification and Chemical Structures of Flavonoids

**Figure 1:**
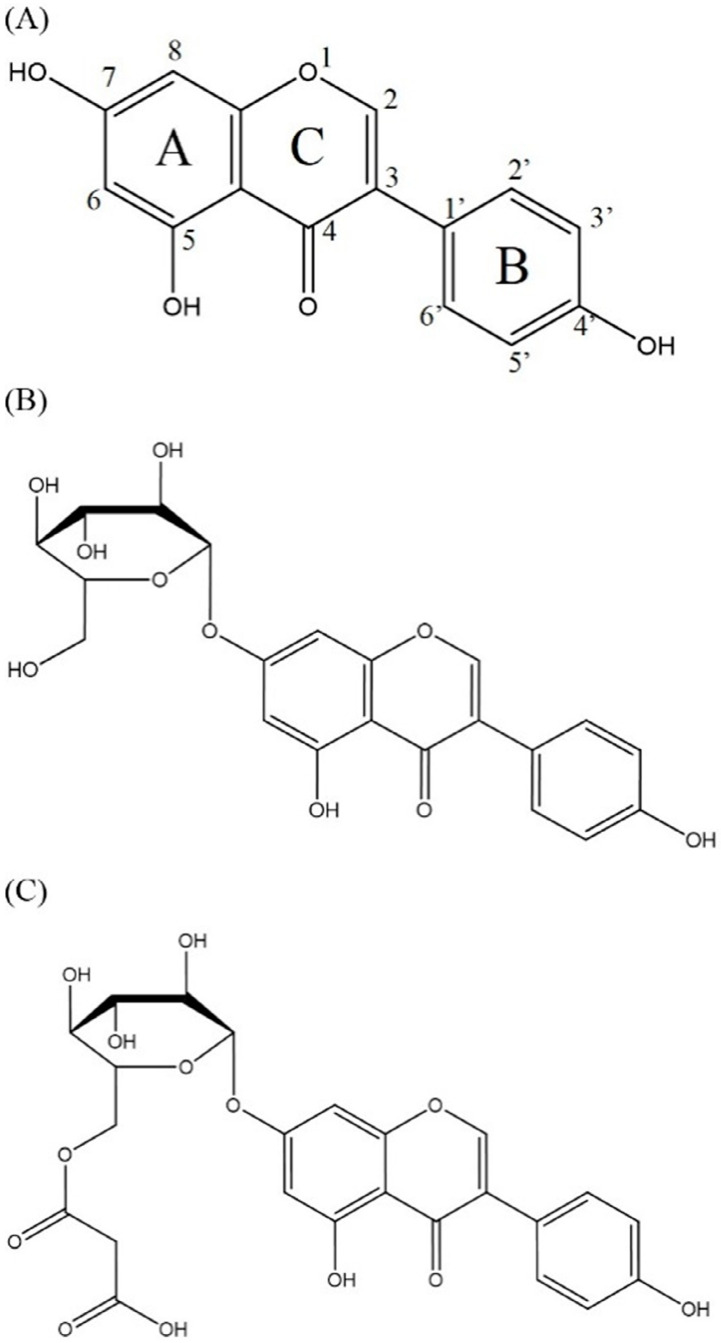
The molecular structure of genistein in (**A**) aglycone form (genistein), (**B**) glycoside form (genistin) and (**C**) conjugated beta-glycoside form (6”-*O*-malonyl genistin).

Flavonoids are a class of naturally occurring polyphenolic compounds which are widely found across the plant kingdom. Flavonoid molecules have a C6-C3-C6 carbon backbone, which is composed of a benzo-γ-pyrone structure and a phenyl ring. The benzene and phenyl ring are denoted as the A ring and B ring respectively, while the oxygen-containing γ-pyrone ring is referred to as the C ring [1] (Figure 1A). Flavonoids can be divided into different sub-groups based on the position at which the B ring is attached to the C ring, and the oxidation status and the degree of saturation of the heterocyclic ring. One distinctive sub-group of flavonoids is isoflavone, which has the B ring attached at position 3 on the C ring, while the other sub-groups, including flavone, flavonol, flavanone, flavanonol, anthocyanidin and flavanol, have the B ring attached at position 2 on the C ring (Figure 1A, Table 1). Structural variations between sub-groups and among members of each sub-group give rise to their functional diversity. The basic structure of a flavonoid is the aglycone form (Figure 1A). However, naturally occurring flavonoids are often modified enzymatically through processes such as hydroxylation, glycosylation, methylation, prenylation, acetylation and sulphation, resulting in numerous derivatives of aglycones with unique biochemical characteristics [2]. Frequently, hydroxylated sites are observed at positions 2, 3, 5, 7, 3’, 4’ and 5’ (Figure 1A). Glycosylation also occurs frequently in nature, usually at position 3 or 7 (Figure 1A). Common carbohydrates attached include D-glucose, L-rhamnose, D-galactose, D-xylose and L-arabinose [3,4]. Using genistein as an example, the basic molecular structures of this flavonoid in the aglycone, glycoside and conjugated β-glycoside forms are illustrated in Figure 1. The general molecular structures of each sub-group of flavonoids are listed in Table 1.

### 2.1. Flavone

Flavones comprise one of the largest sub-groups of flavonoids. The general characteristic of flavones is the presence of a double bond between the carbons at positions 2 and 3, and a ketone group at position 4 on the C ring. There are over 400 types of aglycone flavones, and around 500 *O*-glycosyl and 300 C-glycosyl flavones that have been identified. Flavone glycosides have one or more sugars conjugated to the aglycone flavone via a β-glycosidic bond. *O*-glycosidation usually occurs at positions 7, 3’ and 4’, while C-glycosidation happens at positions 6 and 8 [5]. *O*-methylation and acylation further modify flavones and increase their structural diversity [6]. Flavones are abundant in foods such as chamomile, parsley, celery, cabbage, carrot, celery and wheat sprouts [1].

### 2.2. Flavonol

Flavonols share a similar structure with flavones, except that there is an extra hydroxyl group substituted at C-3. Flavonols seldom occur in the form of C-glycosides, but aglycones (~450 types) and *O*-glycosides (~900 types) are present in most plants. Glycosidic bonds are usually formed at positions 3, 7, 3’ and 4’ [5]. Flavonols are commonly found in fruits and vegetables, such as berries, grapes, tomatoes, onions, kale and broccoli, and plant-based beverages such as red wine and tea [7] (Table 1).

### 2.3. Flavanone

**Figure 2:**
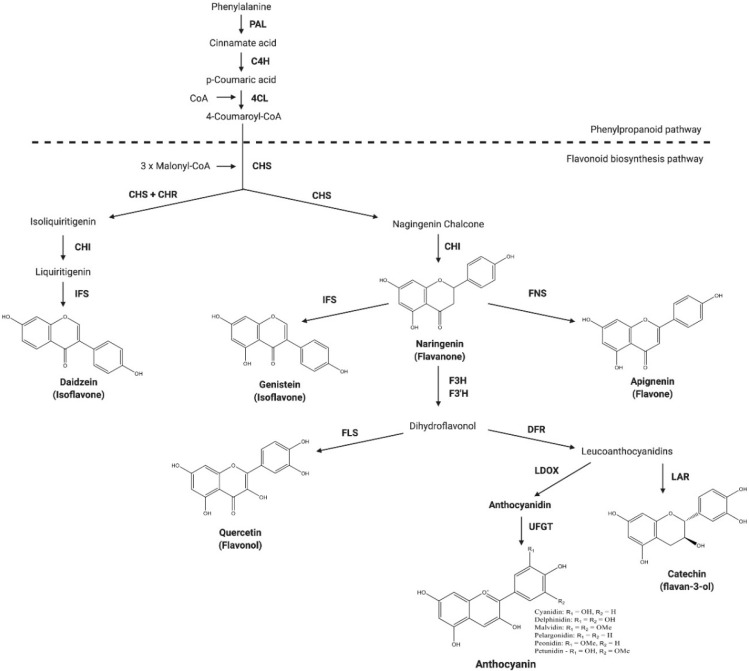
Simplified flavonoid biosynthetic pathway. All classes of flavonoids can be further modified to yield different derivatives with a variety of functions, such as storage and transportation. Malonyl-CoA results from the carboxylation of acetyl-CoA, which is a product of glycolysis and fatty acid β-oxidation. PAL: phenylalanine ammonia-lyase; C4H: cinnamate 4-hydroxylase; 4CL: 4-coumarate-CoA ligase; CHS: chalcone synthase; CHR: chalcone reductase; CHI: chalcone isomerase; IFS: isoflavone synthase; FNS: flavone synthase; FLS: flavonol synthase; F3H: flavanone 3-hydroxylase; F3’H: flavanone 3’-hydroxylase; DFR: dihydroflavonol 4-reductase; LDOX: leucoanthocyanidin dioxygenase; UFGT: UDP-glucose: flavonoid-3-*O*-glycosyltransferase; LAR: leucoanthocyanidin reductase.

Flavanones can be an immediate precursor of other groups of flavonoids, but can also be the end products in the flavonoid biosynthetic pathway (Figure 2). The only structural difference between a flavanone and a flavone is the absence of the double bond between C-2 and C-3, which results in a saturated C ring in flavanones [8]. Flavanones are abundant in citrus fruits such as grapefruit, lemon, lime and orange [9] (Table 1). Flavanones are also the source of the bitter taste in citrus juices [8].

### 2.4. Flavanonol

Flavanonols are the 3-hydroxy derivatives of flavanones. Flavanonols are also referred to as dihydroflavanonols [10]. Taxifolin, a flavanonol, has been known to have a wide range of beneficial pharmaceutical properties, including improving capillary microcirculation, preventing damage to vascular structures of diabetes patients and improving blood flow in the retinal part of the eye [11]. Like flavones, flavonols and flavanones, flavanonols are abundant in citrus fruits [12,13] (Table 1).

### 2.5. Anthocyanidin

The basic structure of anthocyanidins is slightly different from those of the other sub-groups of flavonoids. Anthocyanidins appear as a form of flavylium ion, where the first oxygen atom on the C ring carries a positive charge. Unlike flavonols and flavanones, anthocyanidins lack a ketone group at position 4 on the C ring [1]. Anthocyanidins are unstable and thus are not commonly found in fresh plants. Instead, anthocyanidins occur more frequently in the glycosylated form as anthocyanins, which are a group of water-soluble pigments that contribute to the red, orange, purple and blue colors of plants [1]. Berries, red cabbage, red onion and eggplant are rich in anthocyanins [7] (Table 1).

### 2.6. Flavanol

Flavanols lack a double bond between C-2 and C-3, and the ketone group at position 4 of the C ring. Due to the attachment of a hydroxyl group at position 3 on the C ring, they are also known as flavan-3-ols. This basic structure of flavanol creates two chiral centers on the molecule. Hence, using catechin as an example, there are four diastereoisomers: (+)-catechin, (−)-catechin, (+)-epicatechin and (−)-epicatechin [10]. Red wine, cocoa, tea, apricot, apple, cherry and grape skin are rich in flavanols [14] (Table 1). 

### 2.7. Isoflavone

Among the various sub-classes of flavonoids, isoflavones are widely adopted as health supplements in tablet forms. Legumes, which are major food sources, are rich in isoflavones [15]. The understanding of isoflavone biosynthesis and accumulation in legumes will promote the appropriate consumption of legumes as natural sources of dietary isoflavones.

Isoflavones are derived from 2-phenylbenzypyrone, the basic structure of other flavonoids. Via the aryl-migration mechanism, the attachment of the phenyl ring is moved from position 2 in the flavanone precursor to position 3 on the C ring [1] (Figure 2). Molecules having such basic structure are classified as isoflavonoids. However, the enzyme that converts flavanone to isoflavone is only expressed in several legumes, such as soybean, alfalfa and red clover [15,16]. Among these legumes, soybean is the most abundant source of isoflavones [15] (Table 1). Therefore, soybean seeds are usually regarded as the main food source of isoflavones.

Although isoflavones are only found in several legumes, isoflavones and their derivatives have been extensively researched. Major isoflavones found in nature are daidzein, genistein and glycitein. For storage, isoflavones are commonly modified by *O*-glycosidation [17] to form β-glucosides and 6-*O*-malonylglucosides to be stored in vacuoles. Glycosides are usually further malonylated or acetylated [17]. Glycosylation and malonylation confer the solubility, stability and transportability of isoflavones [17]. In soybean seeds, about 90% of total isoflavones are glucosides [18], with the majority being genistin, daidzin and glycitin, which are the 7-*O*-glucosides of genistein, daidzein and glycitein, respectively. Compared to aglycones, isoflavone glycosides and their glycoconjugates are more water-soluble and therefore suitable for storage in vacuoles [19]. Malonyl-isoflavones appear to be stable and more resistant to enzymatic degradation, thus increasing the retention of the molecules inside vacuoles [19]. Accumulation of these polyphenolic compounds is crucial for plants to respond to microbial infection, herbivory, wounding and abiotic stresses [15]. Plants release isoflavones, such as phytoalexins, as a defense mechanism [15]. Since de-novo biosynthesis of phytoalexins is rate-limiting, studies have revealed an alternative biosynthetic pathway that utilizes stored isoflavone glycosides as precursors [16]. Isoflavones in the form of glycosides are latent, while those in the form of aglycones are biologically active. For example, daidzein can be converted into glyceollins in soybean or phaseollin in french bean, while genistein can be converted to kievitone [15].

Studies indicate that the glycosylation, malonylation and acetylation of isoflavones are important modifications, as the modified molecules not only have enhanced transporter affinity and transportation efficiency, but also improved solubility in water and stability [17]. Previous reports showed that daidzin and genistin, which are isoflavone 7-*O*-glucosides, can be transported into vacuoles via ATP-binding cassette (ABC) transporters or multidrug and toxic compound extrusion (MATE) transporters. MATE2 from *Medicago truncatula* accepts a wide range of substrates and transports different flavonoid glycosides into the vacuole. Nonetheless, malonylated flavonoid glucosides demonstrated the highest transportation efficiency in *M. truncatula* and *Arabidopsis thaliana* [20]. A number of enzymes have been identified in chicken pea, soybean and *M. truncatula* that convert isoflavone 7-*O*-glucosides into malonylated forms. It is highly probable that such a conversion facilitates the transport and storage of the isoflavone molecules [17]. 

## 3. Health Benefits of Flavonoids

### 3.1. The Bioavailability of Flavonoids

Various studies have suggested the potential nutritional and health-promoting effects of flavonoids. The bioavailability of different classes of flavonoids is varied. In general, the most poorly absorbed flavonoids upon ingestion are proanthocyanidins and anthocyanins, while the most readily absorbed flavonoids are isoflavones [24,25]. Ingested flavonoids experience a series of modifications, such as deglycosylation, methylation, glucuronidation and sulphation, along the gastrointestinal tract and circulatory system to yield different conjugates of flavonoids [26,27]. These modifications affect the bioavailability of the flavonoids by affecting their stereochemical configurations and enzyme specificities [25,27,28].

### 3.2. The Health Benefits of Flavonoids: The Molecular Mechanisms

Flavonoids have protective effects against reactive oxygen species (ROS), which are produced as by-products under normal metabolism or induced by extracellular stimuli. ROS play an important role in homeostasis for normal cell functioning. However, the overproduction and accumulation of ROS, known as oxidative stress, upsets the balance between oxidants and antioxidants. Oxidative stress leads to undesirable modifications of lipids, proteins and nucleic acids in the cell, and is associated with various pathological conditions, including diabetes, hepatocellular damage, atherosclerosis and cancers [29,30,31,32,33].

Flavonoids have free radical-scavenging and antioxidative properties. Studies have suggested that the phenolic hydroxyl group on the nucleus of flavonoid molecules contributes to the reducing property of flavonoids [34,35]. Due to the electron donor activities of flavonoids, the direct interactions between free radicals and flavonoid molecules lead to the formation of less reactive radical compounds. Besides this, flavonoids could promote the expression of enzymatic antioxidants such as catalase, superoxide dismutase, glutathione peroxidase and heme-oxygenase in various cell types [36,37,38,39,40]. The induction of non-enzymatic antioxidant synthesis by flavonoids has also been reported in various studies [38,41]. In general, flavonoids help to restore the intracellular oxidative balance by scavenging free radicals and modulating antioxidant enzymes.

Many flavonoids act as phytoestrogens, which have weak interactions with estrogen receptors in mammals and therefore display estrogen-antagonistic effects, reducing the risks of hormone-related cancers, such as endometrial, ovarian, breast and prostate cancers [42,43,44,45]. Epidemiological studies have revealed the negative correlation between the incidence of hormone-related cancer and the intake of phytoestrogen. Countries with high phytoestrogen consumption in their diets were found to have low incidences of hormone-related cancers. Thus, it is believed that flavonoids are potential therapeutic and chemo-preventive agents [42,46,47]. Genistein, which is an isoflavone, is one of the best studied phytoestrogens which targets multiple cellular signal transduction pathways controlling cell cycle regulation and apoptosis [48]. Genistein, which is structurally similar to 17β-estradiol, has been shown to be able to act as the ligand for the estrogen receptor (ER) [42,45,49]. ER undergoes a conformational change after ligand binding, allowing the receptor to interact with chromatin and to modulate the transcription of target genes [42]. The function of genistein as a therapeutic agent in prostate cancer has been evaluated [49]. It was found to act as the demethylating agent for the hypermethylated ER-β promoter in cancer cells, thus alleviating the inhibition of *ER*-*β* transcription. Application of genistein increases the levels of *ER*-*β* mRNA and protein in prostate cancer cells. Furthermore, recent studies have suggested that ER-β modulates autophagy and inhibits cell proliferation in various types of cancers [50,51,52], boosting the potential of flavonoid consumption as a form of chemoprevention and therapy.

Phytoestrogens, particularly soy isoflavones, might play a role in bone density protection due to their estrogen-antagonistic property. It has been suggested that isoflavones might act as anti-resorptive agents in preventing osteoporosis, as observed in vitro, and in clinical and animal tests. Application of the soy isoflavone helped bone formation and maintenance, suppressed osteoclast formation and induced osteoblast formation [53,54,55,56]. However, the capacity of soy isoflavone treatment to prevent menopausal or ovariectomy-induced bone loss is controversial among different research groups. It has been argued that the benefits of isoflavone treatment on human bone density might be biased by the experimental designs, such as the concentrations of the isoflavone applied, treatment durations, sample sizes, types of bone measured, and synergistic effects between isoflavones and other components in the patients’ diets [23,57,58,59]. 

Studies have suggested the anti-inflammatory potential of flavonoids. Flavonoids are effective in inhibiting the edema response in acute inflammation and granulomatous tissue formation in chronic inflammation [60]. In mice, the consumption of flavonoids has been shown to inhibit the expression of proinflammatory cytokines, such as tumor necrosis factor-α and interleukin-6, and pro-inflammatory enzymes, such as cyclooxygenase-2 (COX-2) and inducible nitric oxide synthase (iNOS) [61,62,63,64]. The lowered levels of proinflammatory cytokines and enzymes are possibly associated with the prevention of over-stimulating the inflammatory responses. The use of non-toxic plant-derived flavonoids has been suggested to be a safer alternative to both nonsteroidal anti-inflammatory drugs (NSAIDs) and steroidal anti-inflammatory drugs (SAIDs). Flavonoids have also been suggested to be a safe potential therapeutic and chemo-preventive agent against aging-related disorders, such as rheumatoid arthritis [65]. 

Besides the flavonoid itself, the metabolites resulting from the degradation of flavonoids also possess potential health-promoting effects. For example, it was reported that 3,4-dihydroxyphenylacetic acid (3,4DHPAA), the metabolite from rutin, which is a flavonol glycoside, demonstrated antiproliferative activity in prostate and colon cancer cells [66]. Recently, it was found that 2,4,6-trihydroxybenzoic acid (2,4,6-THBA), the oxidized product of 2,4,6-trihydroxybenzaldehyde (2,4,6-THBAld), which results from the degradation of flavonoids, exhibited inhibitory activities towards cyclin dependent kinase (CDK) and cancer cell proliferation [67]. 

## 4. Regulation of Flavonoid Biosynthesis

The flavonoid contents in plants are tightly regulated by a complex network of regulators. Transcription factors (TFs), including members of the MYB, bHLH, MADS, WRKY and WD40 families, play important roles in regulating flavonoid biosynthesis in plants. These transcription factors may form regulatory complexes. For example, complexes of MYB-bHLH-WD40 (MBW) have been suggested in plants such as *A. thaliana*, *Camellia sinensis*, *Fragaria × ananassa* and *Vitis vinifera* in order to fine-tune flavonoid levels [68,69,70,71,72]. The various combinations of the components in the MBW complex lead to yet another level of regulation in the flavonoid biosynthesis pathways. In general, plant species share common regulators involved in the early steps of the flavonoid biosynthesis pathway. However, different regulators are involved in the regulation of biogenesis genes in the later part of the biosynthesis pathways to determine the specific flavonoids produced and their levels in different plant species [68,73,74,75,76].

### 4.1. Transcriptional Regulation of Flavonoid Biosynthesis

The R2R3-type MYB TFs, which have two repeats (R2R3) of the MYB-type helix-turn-helix domain, are the major group of MYB TFs found in plants with a DNA-binding motif [77]. Plant MYBs are well studied for their involvement in secondary metabolite metabolism, cellular morphogenesis regulation and signal transduction pathway mediation. Various experiments have demonstrated that an R2R3-type MYB is important regulator in the flavonoid biosynthesis pathway. For example, using a transient reporter assay in *G. max*, GmMYB29 was shown to activate the *isoflavone synthetase 2* (*IFS2*) promoter and the *chalcone synthase 8* (*CHS8*) promoter. In *GmMYB29*-overexpressing soybean roots, flavonoid biosynthesis genes, such as *phenylalanine ammonia*-*lyase* (*PAL*), *4*-*coumarate*-*CoA ligase* (*4CL*), *CHS8*, *chalcone reductase* (*CHR*) and *IFS2*, had increased expressions along with elevated isoflavone levels confirmed by high-performance liquid chromatography (HPLC) [74]. The homolog of GmMYB29, LjMYB14, regulates isoflavonoid biosynthesis in *Lotus japonicus* [73]. In blueberry, MYBA, a R2R3-type MYB homolog, was found to be involved in anthocyanin production and accumulation by transactivating the promoter of *dihydroflavonol 4*-*reductase* (*DFR*), the key gene for anthocyanin biosynthesis [75]. On the other hand, the R2R3-type MYBs might also act as negative regulators in the flavonoid biosynthesis pathway. An example is GmMYB183, which promotes the transcriptional level of *GmCYP81E11*, leading to the accumulation of a monohydroxy B-ring flavonoid, which is a less effective anti-oxidative agent compared to a dihydroxy B-ring flavonoid [76]. 

### 4.2. Enzymatic Regulation of Flavonoid Biosynthesis 

The biosynthesis of flavonoids is regulated by specific enzymes. Several groups of plants are famous for being rich in a specific group of flavonoids. For example, berries and grapes are major food sources of anthocyanins, and tea plants are rich in catechins, while soybean is rich in isoflavones. Using these plants as examples, the roles of enzymes in regulating the biosynthesis of these flavonoids will be discussed below.

### 4.3. Anthocyanins 

In plants, anthocyanins are abundantly found in berries and grapes, mainly accumulating in the petal, flower and fruit skin. Fruit skin is the major source of dietary anthocyanin. The importance of DFR in anthocyanin accumulation has been demonstrated in several crops. DFR is the first key enzyme involved in anthocyanin biosynthesis, converting dihydromyricetin into the precursor of anthocyanidins: leucidelphindin (Figure 2). In *Capsicum annuum*, it was found that the expression of *CaDFR* was upregulated during the fruit ripening stage, but was then downregulated afterwards to enable the accumulation of anthocyanins [78]. The gene expression and metabolite profiling of the developing fruit of *Vaccinium corymbosum* (*V. corymbosum*) indicated the transcriptional regulation of flavonoid metabolism and the activation of abscisic acid metabolism [79]. In *V. corymbosum*, the expression level of *VcDFR* was found to be high at the early developmental stages, S1 and S2, followed by a dramatic decrease from the S5 stage to the late stages of S6 to S8, which is the stage when fruits mature, coinciding with the accumulation of anthocyanins [79]. In *Ipomoea batatas* Lam, it was demonstrated that the expression pattern of *IbDFR* correlated with the accumulation of anthocyanins in leaves. Moreover, the overexpression of *IbDFR1* in the Arabidopsis *tt3* mutant, which is deficient in brown tannins in the seed coat, led to the restoration of the pigment [80]. 

### 4.4. Catechins 

Catechins are polyphenols stored in vacuoles [81]. They are the major polyphenols in *C. sinensis,* and account for more than 10% of the polyphenols in dry tea leaves [82]. After entering the phenylpropanoid pathway, catechin biosynthesis involves CHS1, CHS2, CHS3, anthocyanidin synthase (ANS), anthocyanidin reductase 1 (ANR1), anthocyanidin reductase 2 (ANR2) and leucoanthocyanidin reductase (LAR) in leaves at different developmental stages [82]. It was found that the expression level of *CHS1* was highly correlated with the total catechin and epicatechin gallate (ECG) contents, indicating that it plays an important role in catechin accumulation. Besides this, it was shown that the expression levels of *ANR1* and *ANR2* were highly correlated with total catechin, epigallocatechin gallate (EGCG) and ECG contents, indicating their importance for EGCG and ECG biosynthesis. The expression level of *ANS* was also highly correlated with catechin (C) content, indicating its possible role in C biosynthesis. The expression level of *LAR* was highly correlated with ECG and total catechin contents, indicating its possible role in the conversion of C to epicatechin (EC) [82].

### 4.5. Isoflavones 

Isoflavones are uniquely present in several legumes, such as soybean, alfalfa and clover. Among these legumes, soybean is the main protein source in the human diet. The major isoflavones in soybean seeds are genistin, daidzin and glycitin [83]. Isoflavones are synthesized through the phenylpropanoid pathway [83], where *IFS1* and *IFS2* were found to be the essential enzymes for converting the flavanones, naringenin and liquiritigenin, to the isoflavones, genistein and daidzein, respectively [84]. The expressions of genes involved in the phenylpropanoid pathway, chalcone isomerase (*CHI*)*, CHR, IFS1* and *IFS2*, have been studied in different tissues. *CHI*, *CHR* and *IFS1* were found to be expressed in all of the tissues tested, including pod, seed, embryo, flower bud, pod wall, seed coat, flower, leaf, stem and root, while *IFS2* could be detected in all the above-mentioned tissues except pods at 5 and 10 days after flowering (DAF), and seed coats at 30 and 40 DAF [85]. The isoflavone contents in mature seed, leaf and embryo tissues were the highest compared to other tissues [85]. By correlating the gene expression levels with the isoflavone level, *IFS1* and *IFS2* were reported to be the key genes regulating the isoflavone level. Moreover, it was shown that by ectopically expressing *IFS1* from soybean, the non-legume plant *A. thaliana* could be engineered to produce isoflavones [84]. 

### 4.6. Flavonoid-Regulating Genes Are Responsive to Environmental Factors

The flavonoid levels in plants are affected by environmental factors. It has been demonstrated that illumination can induce the expressions of *anthocyanin 1* (*CsAN1*) (an R2R3-type MYB TF), *GLABRA3* (*CsGL3*) and *ENHANCER OF GLABRA3* (*CsEGL3*) (bHLH TFs) in *C. sinensis* [86,87]. Multiple *cis*-acting elements, including light-responsive and phytohormone-responsive elements, were found in the promoter region of *CsAN1*. Long photoperiod induces demethylation of the *CsAN1* promoter, and eventually promotes the expression of the downstream flavonoid regulators, *CsGL3* and *CsEGL3,* and biosynthetic genes for the promotion of anthocyanin production [86]. In *V. corymbosum*, the accumulation of anthocyanin in the fruit after illumination treatment was reported [88,89]. The upregulation of anthocyanin-related transcription factors, *B*-*box protein* (*VcBBX*), *VcMYB21* and *VcR2R3MYB*, was observed after UV-B illumination, followed by the increased expression of late biosynthetic genes including *VcDFR*, *VcANS* and UDP-glucose:flavonoid 3-*O*-glucosyltransferase (*VcUFGT*), resulting in a substantial accumulation of anthocyanins. A similar trend of isoflavone accumulation was observed in soybean sprouts after illumination [90,91]. Genes related to isoflavone biosynthesis, such as *GmCHS*, *flavanone 3*-*hydroxylase* (*GmF3H*), *flavonoid 3’*-*hydroxylase* (*GmF3’H*), *GmIFS* and *isoflavone 6*-*O*-*methyltransferase* (*GmIOMT*) had elevated expression after illumination. Other environmental stimuli, such as low temperature, might also induce the accumulation of flavonoid in plants, including *C. sinensis*, *A. thaliana*, *Citrus sinensis* and *Vitis labrusca* [86,92,93,94]. The trend of flavonoid accumulation upon environmental stimulation, such as light stress, might suggest a protective mechanism shared by different plant species in response to the external stimulus. 

## 5. Accumulation of Flavonoids in Plants due to Domestication

### 5.1. Enrichment due to Artificial Selection

Different food crops are subjected to different selection pressures and criteria based on their degrees of significance in the human diet. Such selection of desirable agronomic traits has possibly contributed to the selection of genes governing the accumulation of different flavonoids in different plant species. For example, agronomic traits such as flavor and environmental adaptability are some of the common targets of selection during domestication. In *G*. *max*, a class B heat shock factor, HSFB2b, known to promote flavonoid accumulation, was selected for during soybean domestication through the selective breeding of individual soybean plants that were better adapted to salt stress [95]. HSFB2b improves the salt tolerance of *G*. *max* by inhibiting the function of the negative regulator of the flavonoid biosynthesis pathway, GmNAC2, a transcription factor, thus activating flavonoid biosynthesis genes. Based on promoter and ChIP-Seq analyses, HSFB2b and GmNAC2 may interact with the promoter regions of the flavonoid biosynthetic genes, including *GmCHS1*, *GmCHS4*, *flavonol synthase 1* (*GmFLS1*) and *flavonol synthase 2* (*GmFLS2*), thus affecting their expression levels and eventually the flavonoid levels in *G*. *max*. The activities of the promoter haplotypes of GmHSFB2b were compared using luciferase reporter assays. The results implied that the promoter haplotype II of GmHSFB2b might have been under heavy positive selection during the domestication of the soybean. 

Many *Camellia* species are consumed as a drinking tea. *Camellia* species exhibited strong artificial selective footprints from the domestication of these tea accessions, as discovered through the study of single nucleotide polymorphisms (SNPs) between wild and cultivated accessions [96]. The flavonoid contents of *Camellia* species might have also been under selection during domestication. For example, the varying ratios between caffeine and catechin determine the flavor of the tea. Using HPLC analyses, a significantly higher total catechin content was revealed in the cultivated accession than in the wild relative [96]. Similar HPLC analyses have shown higher caffeine and catechin contents in *Camellia sinensis* than in the other *Camellia* species [97], resulting in the unique flavor of tea, and thus have been selected for by the breeders of tea plants. Differential expression patterns of metabolic genes, including those regulating caffeine and catechin contents, were observed among different *Camellia* species despite the shared presence of catechin and caffeine biosynthetic genes in their genomes. For example, in the study of tea cultivars pigmented by anthocyanin, the hypomethylation of a CpG island in the *CsAN1* promoter was observed through bisulfite sequencing [86]. Under low temperature and long illumination, demethylation was induced. The resulting increased expression of *CsAN1* specifically upregulated the bHLH TF, CsGL3, which recruited a WD-repeat protein, CsTTG1, that interacted with CsGL3 and CsEGL3 to form the MYB-bHLH-WDR (MBW) complex which is associated with anthocyanin accumulation regulation. Moreover, the co-expression of *CsAN1* and *CsGL3* showed a synergistic effect on the increase in expression of anthocyanin late biosynthetic genes (LBGs), leading to the purple foliage coloration in some tea cultivars [86].

### 5.2. The Exceptional Cases

Not all the agronomic traits are under positive selection for flavonoid contents during domestication. In the studies on *Vaccinium* species, statistically significant interactions among genotype, environment and growing season for both total phenolic and anthocyanin contents were discovered [98,99]. Another study on the anthocyanin contents of different cultivars of blueberries around the world found that genetic influences were found not to play a significant role in determining either total phenolic or anthocyanin contents [100]. Instead, the flavonoid contents were found to be influenced by environmental and geographical factors. It was suggested that the blueberry cultivars available in markets now might have been subjected to specific agronomic and fruit trait selections that were not necessarily related to the abundance of health-promoting phytochemicals in the fruit. The previous breeding programs of blueberry cultivars might have selected for highly heritable traits other than anthocyanin contents [94,96].

## 6. Transport of Flavonoids

Flavonoids are found both intracellularly and extracellularly in plants. Inside plant cells, flavonoids are distributed in various compartments, including nuclei, ERs, vacuoles, vesicles and chloroplasts. In soybean, it has been reported that isoflavones are stored in vacuoles in the glycosylated or malonylated form [101]. On the other hand, in pea, it was found that isoflavone synthase, CYP93C18, is localized in the ER [102]. These patterns of intracellular storage and biosynthesis locations highlight the significance of flavonoid transport in influencing the storage and the bioavailability of flavonoids.

The transportation of flavonoids can be facilitated by vesicles or transporter proteins. One of the transporter proteins is glutathione-S-transferase (GST). GST acts as a carrier protein by binding to flavonoids. In soybean, the binding of a putative lambda class GST, GmGSTL1, to flavonoids was reported [103]. ATP-binding cassette (ABC) transporters and multidrug and toxic compound extrusion (MATE) transporters have been characterized in various plant species as the transporters of flavonoids [104]. In general, transporter proteins are integral membrane proteins which facilitate the movement of molecules across membranes [105]. The specific protein domains of ABC transporters and MATE transporters which confer their functions will be discussed below. These two types of transporters mediate the secretion or the accumulation of flavonoids in plants. Since the mechanisms of secretion and uptake of flavonoids by plant cells have been discussed in previous reviews [104,106,107], this review will discuss the transportation of flavonoids from the perspective of the nutritional contents of plants for human consumption; in other words, the types of transportation that can facilitate the accumulation of flavonoids in the edible portion of crop plants.

### 6.1. Vesicle-Mediated Accumulation of Flavonoids

In maize, using transgenic Black Mexican Sweet (BMS) cells as a model, the accumulation of anthocyanin in vesicles was observed [108]. The anthocyanin-containing vesicles eventually merged to form a single vacuole [108]. In *A. thaliana*, it was reported that mutants with a mutated GFS9, a membrane trafficking factor, were defective in vesicle trafficking and had low anthocyanin accumulation in the seed coat [109]. In some occasions, the vesicle-mediated transportations are assisted by GST. In *Vigna radiata*, the transportation of medicarpin conjugated with GSH (the reduced form of glutathione) into vacuolar vesicles was reported [110]. In *V. vinifera*, it was found that transgenic hairy roots with knocked-down *VvGST* were not able to accumulate anthocyanins in vacuoles, although anthocyanins were found in vesicles [104]. 

### 6.2. ABC Transporter-Mediated Accumulation of Flavonoids

ABC transporters are characterized by their conserved nucleotide-binding domains (NBDs). The conserved motifs include the Walker A motif, Walker B motif, Q-loop, D-loop and the LSGGQ motif, which is exclusively found in ABC transporters but not other ATPases [111]. ABC transporters are categorized into three sub-types: full transporters, half transporters and transporters without transmembrane domains (TMDs). The full transporters consist of two TMDs and two NBDs. The half transporters consist of one TMD and one NBD, and usually exist as dimers in order to become a virtual full transporter. Although the third type of ABC transporters do not contain TMDs, they still have two NBDs [111]. ABC transporters drive the active transport of molecules across membranes by the hydrolysis of ATP [112]. In plants, ABC transporters have been found in the plasma membrane and the tonoplast [112].

**Figure 3:**
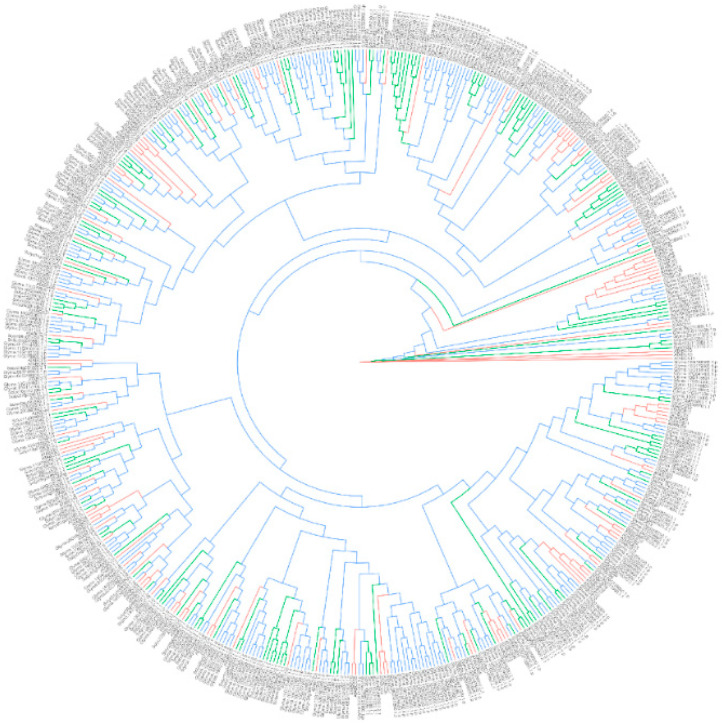
Phylogenetic analysis of ATP-binding cassette (ABC) transporters from *G. max*, *S. lycopersicum* and *A. thaliana*. The amino acid sequences of the ABC transporters identified from *G. max* [115], *S. lycopersicum* [116] and *A. thaliana* [119] were aligned using Clustal Omega [126] with default parameters. The phylogenetic tree was constructed using RAxML [127] with 1000 times rapid bootstrapping. The protein model was selected automatically by the maximum likelihood criterion.

Based on their characteristic protein structures, ABC transporters have been identified in various plant species. Using genome-wide analyses, 91, 160, 261, 154, 100, 129, 120, 135 and 130 putative genes encoding ABC transporters were identified in the genomes of *L. japonicus* [113], *M. truncatula* [114], *G. max* [115], *Solanum lycopersicum* [116], *Ananas comosus* [117], *Oryza sativa* [118], *A. thaliana* [119], *V. vinifera* [120] and *Zea mays* [121], respectively (Table 2). A phylogenetic analysis of the ABC transporters identified from *G. max*, *S. lycopersicum* and *A. thaliana*, the species with available genome information, is shown in Figure 3. Despite the abundance of ABC transporter genes in these plant genomes, only several of the genes have been functionally characterized. In *G. max*, the first biochemical characterization of genistein secretion from roots suggested that such secretion was associated with an ABC-type transporter, although the protein(s) responsible for such secretion has (have) not yet been discovered [122]. In *Z. mays*, ZmMRP3 was found to be localized in the tonoplast and involved in the transport of anthocyanins [123]. In *M. truncatula*, a full ABC transporter, MtABCG10, was reported to be associated with the accumulation of isoflavonoids [124]. The silencing of *MtABCG10* in transgenic roots resulted in the decreased accumulation of medicarpin and its precursors, and increased sensitivity to *Fusarium oxysporum* infection [124]. In *V. vinifera*, ABCC1 was found to be localized in the tonoplast and involved in the transport of anthocyanidin 3-*O*-glucosides [125]. Functionally characterized ABC transporters which are involved in the accumulation of flavonoids in plant cells are summarized in Table 2.

### 6.3. MATE Transporter-Mediated Accumulation of Flavonoids

MATE transporters, typically consisting of twelve transmembrane domains, are cation antiporters which transport a broad spectrum of molecules, including hormones, organic acids and secondary metabolites, across membranes in exchange for Na^+^ or H^+^ ions [103,122,123]. The movement of Na^+^ or H^+^ across the membrane creates an electrochemical gradient, which is the driving force for the transport of substrates [107]. MATE transporters are categorized into three sub-types: NorM and Din F (DNA damage-inducible protein F), which are made up of eubacterial and archaeal proteins, and the eukaryotic subfamilies [128]. Among the three subtypes of MATE transporters, NorM and Din F can transport Na^+^ or H^+^, while eukaryotic MATE transporters transport only H^+^ to create the electrochemical gradient. 

**Figure 4:**
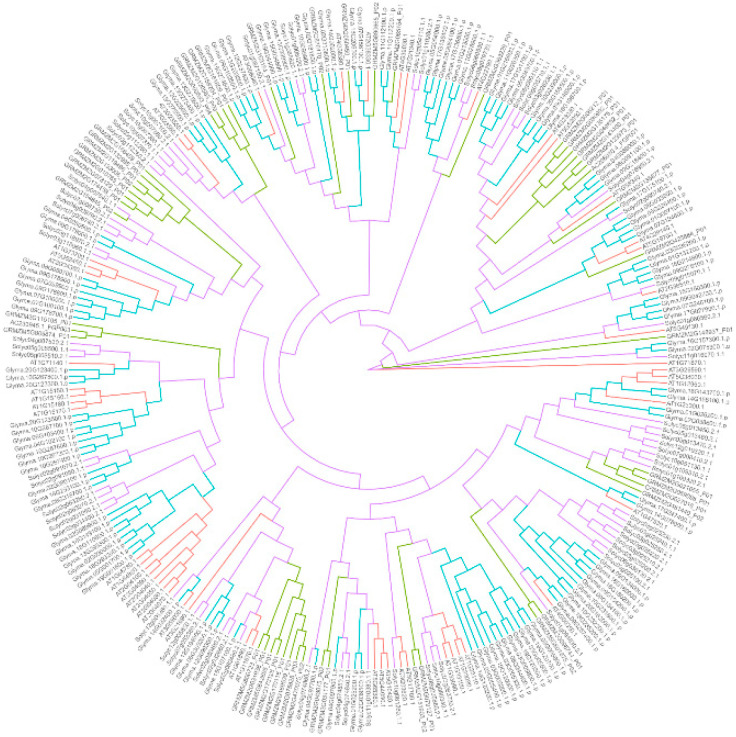
Phylogenetic analysis of multi-antimicrobial extrusion protein (MATE) transporters from *G. max*, *Z. mays*, *S. lycopersicum* and *A. thaliana*. The amino acid sequences of the MATE transporters identified from *G. max*, *Z. mays*, *S. lycopersicum* and *A. thaliana* [129,130,131,134] were aligned using Clustal Omega [126] with default parameters. The phylogenetic tree was constructed using RAxML [127] with 1000 times rapid bootstrapping. The protein model was selected automatically by the maximum likelihood criterion.

Through genome-wide analyses, 117, 49, 67, 71, 53, 56, 40, 65, 48 and 33 putative genes encoding MATE transporters have been identified in *G. max* [129], *Z. mays* [130], *S. lycopersicum* [131], *Populus trichocarpa* [132], *O. sativa* [133], *A. thaliana* [134], *M. truncatula* [135], *V.* *vinifera* [136], *Solanum tuberosum* [137] and *V. corymbosum* [138], respectively (Table 2). The phylogenetic analysis of the MATE transporters identified from *G. max*, *Z. mays*, *S. lycopersicum* and *A. thaliana*, the species with available genome information, is shown in Figure 4. In plants, the first MATE transporter was identified in *A. thaliana* for its role in extruding growth inhibitors from the roots [139]. Besides their role in extrusion, MATE transporters have also been reported to facilitate the sequestration of molecules in vacuoles [129,134,135]. TT12 in *A. thaliana* is a MATE transporter localized in the tonoplast, and is required for the sequestration of flavonoids in vacuoles [140]. *tt12* mutant seeds showed a reduced accumulation of proanthocyanidin (PA) [140]. *TT12* was later found to be identical to *AtDTX41,* which was identified in a genome-wide survey of *MATE* genes in *A. thaliana* [134]. The ectopic expression of *MdMATE1* and *MdMATE2* from apple (*Malus* x *domestica*) in the Arabidopsis *tt12*-*1* mutant improved the accumulation of quercitrin in the seed, although the quercitrin level was still lower than in the wild type Arabidopsis [141]. In *M. truncatula*, MATE1 was found to be localized in the tonoplast and facilitate the vacuolar uptake of epicatechin 3’-*O*-glucoside, which is the precursor of PA [135]. In the same study, it was found that the ectopic expression of *TT12* from *A. thaliana* in *M. truncatula* hairy roots induced the vacuolar transport of epicatechin 3’-*O*-glucoside [135]. Despite their sequence similarity, MATE1 and MATE2 in *M. truncatula* have different substrate specificities. MtMATE2 was found to be localized in the tonoplast, and to mediate the vacuolar sequestration of flavonoid glycosides, including anthocyanins and flavone glucosides [20]. In *V.* *vinifera*, anthoMATE1 and anthoMATE3 were found to be localized in the tonoplast, and to mediate the transport of acylated anthocyanins to be stored in the vacuole [136]. Besides anthoMATE1 and anthoMATE3, VvMATE1 and VvMATE2 were also characterized from *V.* *vinifera*. However, unlike anthoMATE1 and anthoMATE3, it was suggested that VvMATE1 and VvMATE2 are involved in the transport of PA [142]. Functionally characterized MATE transporters which are involved in the accumulation of flavonoids in plant cells are summarized in Table 2. However, although soybean is a major source of isoflavones in the human diet, its MATE transporters are underexplored. A recent study of quantitative trait loci (QTLs) revealed an overlapping QTL of soybean seed antioxidants, phenolics and flavonoids [143]. In this QTL, there are two putative transporter proteins: GmMATE1 and GmMATE2 [143]. Adjacent to the QTL, there is also one putative MATE transporter: GmMATE4 [143]. 

Numerous putative genes encoding ABC transporters or MATE transporters have been identified in model plants, legumes and berries (Table 2). However, only a small portion of the identified genes have been functionally characterized. ABC transporters and MATE transporters have been demonstrated to transport flavonoids (Table 2). Previous research has demonstrated the diverse substrate specificities of these transporters. Considering the numerous uncharacterized ABC transporters and MATE transporters, there are lots of opportunities for related research. In this review, we provided the phylogenetic analyses of putative ABC transporters and MATE transporters (Figure 3 and Figure 4) and summarized those transporters that have been functionally characterized (Table 2), so as to provide a direction for future research on the functions of these transporters.

## 7. Future Direction of Flavonoid Research on Crops

The accumulation of flavonoids in crops is one of the determining factors controlling the bioavailability of flavonoids to the human body. Equally important is the digestion and absorption of the flavonoids in the human body. Flavonoids are chemically unstable and are known to be degraded by gut microbes upon ingestion [144,145]. Besides gut microbes, the intestinal juices of different pH values also mediate the auto-degradation of flavonoids [146]. A high rate of the degradation of flavonoids, which results in the production of phenolic acids [147], may reduce the absorption efficiency of the flavonoids by the human body [144]. However, there is increasing evidence suggesting the possible health-promoting effects of such phenolic acid, as discussed above. It has also been proposed that the phenolic acids resulting from the degradation of flavonoids and those resulting from the degradation of aspirin prevent cancer by a common pathway [148]. Currently, the fates of flavonoids upon ingestion and the roles of metabolites resulting from degraded flavonoids in the gut are largely unknown. More research will be needed to fully understand the bioavailability and health benefits of flavonoids and their degraded products from crops.

## 8. Conclusions

Common crops for human consumption, including fruits, vegetables and legumes, are rich in different forms of flavonoids. Tea, which is an important plant for making beverages in a lot of Asian countries, is abundant in catechin, a flavanol shown to have health benefits. Flavonoid molecules have similar yet different structures. The subtle molecular mechanisms in the regulation of their biosynthesis bring forth a vast variety of flavonoids and their derivatives. The different molecular structures of flavonoids give rise to the different functions of these molecules. Equally important is the transport of flavonoids in the regulation of their contents in plant cells and thus their availability for human consumption. The understanding of the biological functions of flavonoids and the molecular mechanisms regulating their abundances in food crops will facilitate the smart use of crops in our diet and enable breeding programs to produce crops with desirable contents of flavonoids.

## Figures and Tables

**Table 1:** Molecular structures, examples and food sources of flavonoids.

Sub-Class of Flavonoids	Example Structure(s) of Aglycone Flavonoids	Examples	Food Sources	References
Flavone	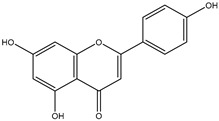 Apignenin	Apigenin and luteolin	Chamomile, parsley, celery, cabbage, carrot, celery, wheat sprout, citric fruits	[1,21]
Flavonol	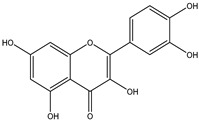 Quercetin	Quercetin, kaempferol or myricetin	Berries, apple, grapes, tomatoes, onion, kale, broccoli, tea, red wine, olive oil, citric fruits	[7]
Flavanone	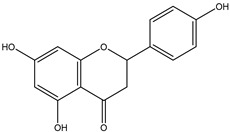 Naringenin	Hesperetin, naringenin and naringin	Citrus fruits	[9]
Flavanonol	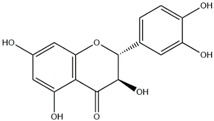 Taxifolin	Taxifolin	Citrus fruits, tea, rice	[11]
Anthocyanidin	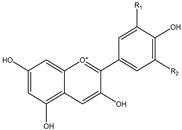 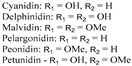	Cyanidin, pelargonidin, delphinidin, malvidin, petunidin and peonidin.	Black currant, blueberry, cherry, elderberry, grapes, red cabbage, red onion, eggplant	[7,22]
Flavanol	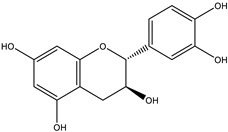 Catechin	Catechin, epicatechin, gallo-catechin	Apricot, apple, cherry, grape (skin), cocoa, tea, red wine	[14,23]
Isoflavone	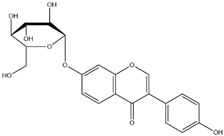 Daidzin 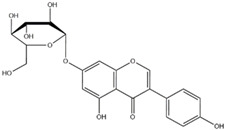 Genistin 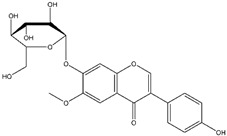 Glycitin	Daidzin, genistin and glycitin	Soybean, red clover, alfalfa, kudzu	[1,15]

**Table 2:** Summary of ABC transporters and MATE transporters identified in different plant species.

Transporter	Species	Number of Putative Genes Identified in the Genome	Functionally Characterized Genes Involved in the Accumulation of Flavonoids	Substrates	References
ABC transporter	*L. japonicus*	91			[113]
	*M. truncatula*	160	MtABCG10	Medicarpin and its precursors	[114,124]
	*G. max*	261			[115]
	*S. lycopersicum*	154			[116]
	*A. comosus*	100			[117]
	*O. sativa*	129			[118]
	*A. thaliana*	120			[119]
	*V.* *vinifera*	135	ABCC1	Anthocyanidin 3-*O*-glucosides	[120,125]
	*Z. mays*	130	ZmMRP3	Anthocyanin	[121,123]
MATE transporter	*G. max*	117			[129]
	*Z. mays*	49			[130]
	*S. lycopersicum*	67			[131]
	*P. trichocarpa*	71			[132]
	*O. sativa*	53			[133]
	*A. thaliana*	56	TT12	PAs, anthocyanin, epicatechin 3’-*O*-glucoside	[135,140]
	*M. truncatula*	40	MATE1	Epicatechin 3’-*O*-glucoside	[135]
			MATE2	Anthocyanin, flavone glucosides	[20]
	*V.* *vinifera*	65	anthoMATE1	Acylated anthocyanins	[136]
			anthoMATE3	Acylated anthocyanins	[136]
			VvMATE1	PAs	[142]
			VvMATE2	PAs	[142]
	*S. tuberosum*	48			[137]
	*V. corymbosum*	33			[138]
